# Hydrostatic pressure activates HIF‐1α via β‐catenin to promote stemness in breast cancer cells

**DOI:** 10.1002/2211-5463.70267

**Published:** 2026-06-01

**Authors:** Da Zhai, Yong Xu, Jingjing Ma, Ratchanee Duangrat, Chen Yan, Jingyan Liang, Hua Wei, Bin Jiang, Tao‐Sheng Li

**Affiliations:** ^1^ Department of Rheumatology and Immunology Northern Jiangsu People's Hospital Yangzhou China; ^2^ Department of Stem Cell Biology Atomic Bomb Diseases Institute, Nagasaki University Japan; ^3^ Department of Stem Cell Biology Nagasaki University Graduate School of Biomedical Sciences Japan; ^4^ Department of Zoology, Faculty of Science Kasetsart University Bangkok Thailand; ^5^ Institute of Translational Medicine, Medical College Yangzhou University Jiangsu China; ^6^ Colorectal Disease Center of Nanjing Hospital of Chinese Medicine Affiliated to Nanjing University of Chinese Medicine Jiangsu Province China

**Keywords:** ALDH, HIF‐1α, hydrostatic pressure, stemness, β‐catenin

## Abstract

Tumor microenvironment plays a pivotal role in regulating the biological properties of cancer cells. Interstitial fluid hydrostatic pressure (HP) generally increases in malignant solid tumors. While our recent work has demonstrated that elevated HP stabilizes HIF‐1α expression in cancer cells, here we further investigate whether elevated HP promotes cancer cells to acquire stemness features. Our results showed that exposure to 50 mmHg HP for 48 h did not significantly change the survival or morphology of human breast cancer cells. However, 50 mmHg HP stimulation significantly increased aldehyde dehydrogenase (ALDH) activity and upregulated HIF‐1α and stemness markers (CD44 and SSEA‐1) in MCF‐7 and BT‐474 but not in MDA‐MB‐453 breast cancer cell lines. Correspondingly, β‐catenin was significantly enhanced in MCF‐7 and BT‐474 breast cancer cell lines under 50 mmHg HP stimulation and was negatively expressed in MDA‐MB‐453 breast cancer cells. Hypoxia (1% O_2_) exposure for 48 h also significantly enhanced the expression of β‐catenin only in MCF‐7 cells and BT‐474 breast cancer cell lines, but increased HIF‐1α expression and ALDH activity in all breast cancer cell lines tested, including the β‐catenin‐deficient MDA‐MB‐453 cells. Therefore, we conclude that elevated HP in malignant tumors may facilitate the acquisition of ‘stemness’ characteristics of cancer cells by enhancing HIF‐1α expression via β‐catenin.

## Graphical abstract

To mimic the elevated intestinal fluid pressure in breast cancers, we loaded human breast cancer cells (MCF‐7, MDA‐MB‐453, and BT‐474) to 50 mmHg hydrostatic pressure. Hydrostatic pressure exposure upregulated HIF‐1α and induced stemness in MCF‐7 and BT‐474 cells. In contrast, β‐catenin‐deficient MDA‐MB‐453 cells were found poorly responsive to hydrostatic pressure stimulation.

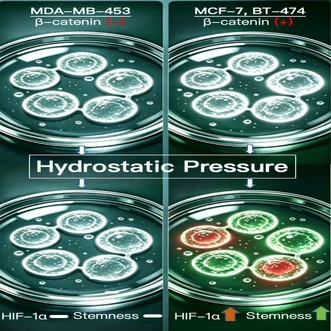

**Abbreviations**
3D: three dimensional
ALDH: aldehyde dehydrogenases
CON: control
CSCs: cancer stem cells
DAPI: 4, 6‐diamidino‐2‐phenylindole
ECM: extracellular matrix
HIF‐1α: hypoxia‐inducible factor 1‐alpha
HP: hydrostatic pressure
Hypo: hypoxia
PDX: patient‐derived xenograft
Wnt signaling pathway: Wingless/Int‐1 signaling pathway

Cancer progression is known to be associated with the existence of cancer stem cells (CSCs) within the tumor bulk [1, 2, 3]. CSCs, which are defined as cells with tumor‐initiating properties and infinite self‐renewal/proliferative potential, are the most relevant and elusive cancer cell population, as they have the exquisite ability to seed new tumors. CSCs are thought of as ‘queen bees’ who create and proliferate many ‘worker bees’ or nonstem cancer cells. CSCs have been demonstrated to contribute crucially to the prognosis of malignant tumors [4]. Thus, understanding the molecular mechanisms involved in stemness and CSC specification and maintenance is critical for developing new therapeutic strategies and improving overall survival in clinics.

Breast carcinoma is the most frequently diagnosed malignant tumor and the leading cause of cancer‐related death among women worldwide [5]. Aldehyde dehydrogenases (ALDH) are a group of enzymes that catalyze the oxidation of aldehydes, acting as an ‘aldehyde scavenger’ found across species. High ALDH activity has been proposed as a common biomarker of CSCs and is associated with poor cancer prognosis in various malignant tumors, including breast carcinoma [6].

With the rapid advancement of mechanobiology in recent years, it has been demonstrated that biomechanical forces in the tissue microenvironment play critical roles in regulating cell biological behaviors and malignant tumor progression [7, 8, 9]. Beyond the alternation of stiffness due to extracellular matrix (ECM) accumulation, an elevated interstitial fluid hydrostatic pressure (HP) generally occurs in rapidly growing malignant tumors [10, 11]. For example, the interstitial fluid pressure is 4.0–53.0 mmHg in breast carcinoma, but it keeps 0.0–3.0 mmHg in normal breast tissue [12]. Elevated interstitial fluid HP in malignant tumors has been demonstrated to accelerate motility in cancer cells [7] and associates with worse prognosis of patients [13]. It has been reported that HP enhances cellular viability and improves cell differentiation in various kinds of cells [14, 15, 16, 17]. However, the relevant molecular mechanism remains unclear.

Mechanotransduction is a process of translating mechanical stimuli into biochemical signals and performs important regulatory functions. There are multiple pathways for cellular mechanotransduction, including membrane receptor activation, reorganization of the nucleus, and mechanosensitive transcription factors. The process of mechanotransduction can be via ECM, adhesion, ion channels, and so on [18]. β‐catenin, a key molecule in Wnt signaling, has been demonstrated to be associated with mechanotransduction [19, 20] and plays a pivotal role in ECM and adhesion [21]. Wnt/β‐catenin signaling has been demonstrated to play a critical role in CSC self‐renewal [22] and tumor initiation [23] in breast cancer.

HIF‐1 involves multiple processes of tumor progression, such as angiogenesis, ECM remodeling, invasion, and metastasis [24, 25]. HIF‐1 also plays a pivotal role in promoting specification and maintenance of CSCs, and the knockdown of HIF‐1α impairs the tumor‐initiating ability of CSCs [4, 26, 27]. We have recently demonstrated that elevated HP stabilizes HIF‐1α to promote cancer cell metastasis [28]. Therefore, elevated interstitial fluid HP in a rapidly growing malignant tumor may induce HIF‐1α to promote stemness of cancer cells.

To examine our hypothesis, human breast cancer cells were exposed to 50 mmHg HP for 48 h. Then, we investigated the effect and relevant mechanism of HP exposure on the stemness property of breast cancer cells.

## Materials and methods

### Breast cancer cell lines

Three human breast cancer cell lines [MCF‐7 (RRID:CVCL_0031), MAD‐MB‐453 (RRID:CVCL_0418) and BT‐474 (RRID:CVCL_0179)] were used for experiments. MCF‐7, MAD‐MB‐453, and BT‐474 cells were purchased from RIKEN BRC Cell Bank. All three cell lines have been authenticated in the past 3 years by profiling single nucleotide polymorphism or analysis of short tandem repeats. All cells were regularly checked for mycoplasma contamination and all experiments were performed with mycoplasma‐free cells.

The cells were maintained in RPMI‐1640 (Wako), supplemented with 1% penicillin/streptomycin (Gibco; Thermo Fisher Scientific, Inc.) and 10% fetal bovine serum (Cytiva), and cultured in a humidified incubator with 5% CO_2_ at 37 °C.

### HP stimulation and hypoxia treatment

HP stimulation was induced to cells using a pneumatic pressurizing system (Strex. Inc.). Briefly, breast cancer cells were seeded in 60‐mm diameter Petri dishes (3 × 10^5^ cells/dish) and cultured for about 36 h to form an adherent monolayer. Then, the culture dishes were randomly selected to move into a sealed chamber in which 50 mmHg HP was stably kept for 48 h (HP group). Similarly, hypoxia treatment was done by keeping the culture dishes under 1% O_2_ condition for 48 h (Hypo group) using MCO‐5 M incubator (SANYO. Inc.). The culture dishes without HP stimulation nor hypoxia treatment were used as the control (CON group).

### Cell morphology and cell survival

Cell morphology was observed under a light microscope (1X71S8F‐3; Olympus Corporation). The cells were then collected as a single cell suspension. To measure the total cell number, 10 μL cell suspension was mixed with 10 μL trypan blue, and the viable cells were counted using dual‐chamber cell counting slides (catalog no. 1450011; Bio‐Rad) and a TC20™ Automated Cell Counter (Bio‐Rad Laboratories, Inc.).

### ALDH activity assay

ALDH activity was measured using a colorimetric assay kit (cat no.: MAK082; Sigma‐Aldrich). Briefly, cell pellets were homogenized in ice‐cold ALDH assay buffer. The extracted protein concentration was measured using a BCA assay. A total of 200 μg protein at 4 μg·μL^−1^ concentration from each sample was used for detecting. NADH standards for colorimetric detection were also made by adding 0, 2, 4, 6, 8, and 10 μL of the 1 mm standard solution into a 96 well plate, generating 0 (blank), 2, 4, 6, 8, and 10 nmol per well standards. Samples were added to the 50 μL reaction mixture to a final volume of 100 μL. The mixture was mixed well and incubated at room temperature for 5 min. Then the initial absorbance was measured at 450 nm with iMark™ Microplate Reader (Bio‐Rad). The measurement was repeated every few minutes until the reading was greater than the value of the highest standard. The final measurement would be the penultimate reading or the value before the most active sample is near or exceeds the end of the linear range of the standard curve. The calculation of ALDH activity was followed measurement method ALDH activity = B * Sample dilution factor/(reaction time * V), where B = amount (nmole) of NADH generated between T_initial_ and T_final_, Reaction time = T_initial_ − T_final_ (minutes), V = Sample volume (mL) added to well.

### Western blot analysis

The expression of HIF‐1α and β‐catenin was evaluated by western blot, as previously described [29]. Total protein was extracted from cells using 1X RIPA buffer (FUJIFILM Wako Pure Chemical Corporation). The concentration of protein was detected using a BCA assay. A total of 40 μg protein from each sample was separated using 10% SDS/PAGE, then was transferred to 0.2‐μm PVDF membranes (Bio‐Rad Laboratories, Inc.). After blocking with 5% skimmed milk at room temperature for 1 h, the membranes were incubated with primary antibodies against HIF‐1α (1 : 250 dilution; ab1; 2 h at room temperature; Abcam), β‐catenin (1 : 2000 dilution; ab32572; overnight at 4 °C; Abcam), and β‐actin (1 : 1000 dilution; cat. no.: 8457S; overnight at 4 °C; Cell Signaling Technology, Inc.), followed by incubation with horseradish peroxidase‐conjugated secondary antibodies for 1 h at room temperature. Using an enhanced chemiluminescence detection kit (Thermo Fisher Scientific, Inc.), the expression level was visualized. Semi‐quantitative analysis was done using ImageQuant LAS 4000 mini detection system (v1.0; GE Healthcare Life Sciences).

### Immunofluorescence staining

Immunofluorescence staining was performed to detect the protein levels of CD44, SSEA‐1, and filaments of F‐actin. Cells were seeded onto 4‐well culture chamber slides. When growing to around 70% confluence, the cells were exposed to 50 mmHg HP for 48 h as above. The cells were fixed by 4% paraformaldehyde at room temperature for 10 min, and then treated with 0.1% Triton X‐100 at room temperature for another 10 min. After blocking by 3% bovine serum albumin at room temperature for 30 min, the cells were incubated with primary antibodies against CD44 (1 : 200 dilution; ab6124; Abcam) and SSEA‐1 (1 : 800 dilution; 14–8813‐82; Invitrogen) overnight at 4 °C, followed by incubation with a secondary antibody (1 : 500 dilution, Invitrogen) for 1 h at room temperature in the dark. F‐actin fibers were stained with TRITC‐phalloidin in mounting medium (Vectorlabs, Burlingame, CA, USA). The nuclei were stained with 4, 6‐diamidino‐2‐phenylindole (DAPI). Immunofluorescences were detected using an inverted fluorescence microscope (Olympus FV10i; Olympus). For each staining, at least 10 images were taken from randomly selected fields at 60 magnifications, and the mean fluorescence intensity was measured by the IMAGE J software (NIH).

### Statistical analysis

Data are presented as mean ± SD. Statistical significance between two groups was determined using an unpaired *t*‐test (SPSS; v20.0; IBM Corp.). Statistical significance of multi‐group was determined using one‐way ANOVA followed by Tukey's test (SPSS; v20.0; IBM Corp.). *P* < 0.05 was used to indicate a statistically significant difference.

## Results

To test whether HP stimulation promotes stemness of cancer cells, ALDH activity was measured in MCF‐7 cells at 0, 6, 24, and 48 h after the exposure to 0, 50, and 100 mmHg HP. Our data showed that HP stimulation increased the ALDH activity in MCF‐7 cells in force‐ and time‐dependent manners (Fig. 1A,B). As the comparable increase of ALDH activity in MCF‐7 cells under 50 and 100 mmHg HP stimulation and the range of 4.0–53.0 mmHg interstitial fluid HP in breast carcinoma tissues [12], we selected 50 mmHg in the following experiments. We confirmed that 50 mmHg HP exposure for 48 h did not induce notable morphological change (Fig. 1C) and had a limited effect on cell growth (Fig. 1D) in all three cell lines used for the study.

**Fig. 1:**
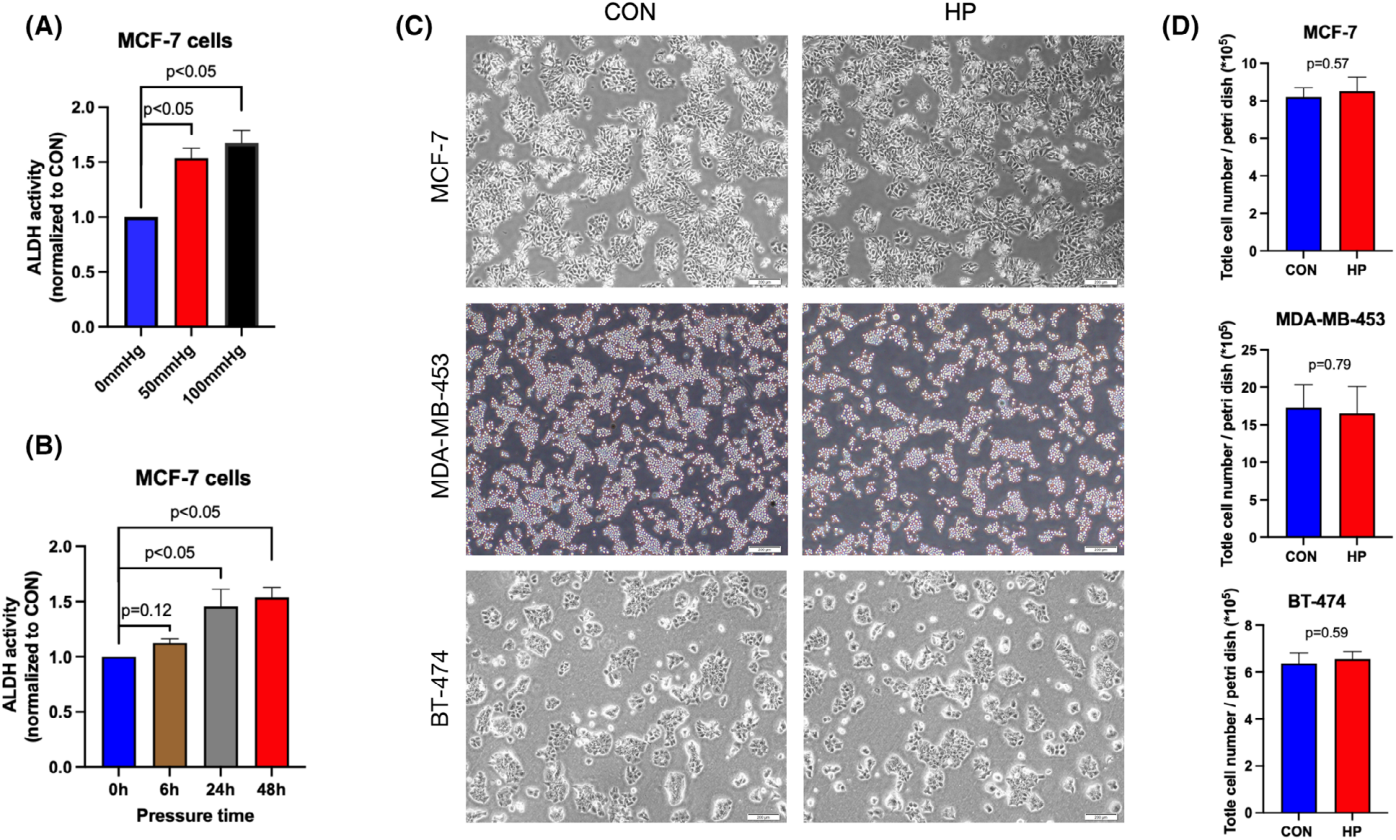
Aldehyde dehydrogenase (ALDH) activity and cell viability of breast cancer cells under hydrostatic pressure (HP) stimulation. (A) ALDH activity of MCF‐7 cells after exposure to 0, 50, or 100 mmHg HP for 48 h. The data are presented as the mean ± SD from three independent experiments and statistical significance was determined using one‐way ANOVA followed by Tukey's test. (B) ALDH activity of MCF‐7 cells after exposure to 50 mmHg HP for different times (0 h, 6 h, 24 h, and 48 h). The data are presented as the mean ± SD from three independent experiments and statistical significance was determined using one‐way ANOVA followed by Tukey's test. (C) Representative images of cell morphology under a phase‐contrast microscope. (D) Quantitative analysis of the total number of surviving cells per petri dish. Scale bar, 200 μm. The data are presented as the mean ± SD from three independent experiments and statistical significance was determined using unpaired *t*‐test. HP, hydrostatic pressure; CON, control.

Interestingly, 48‐h exposure to 50 mmHg HP significantly increased the ALDH activity in MCF‐7 cells (*P* < 0.05 vs. control; Fig. 2) and BT‐474 cells (*P* < 0.05 vs. control; Fig. 2), but was not significantly changed in MDA‐MB‐453 cells (*P* = 0.31 vs. control; Fig. 2). We also performed immunostaining on the expression of stemness markers CD44 and SSEA‐1 in cells. After 48 h exposure to 50 mmHg HP, the expression of CD44 and SSEA‐1 was significantly enhanced in MCF‐7 cells (*P* < 0.05 vs. control; Fig. 3A,B) and BT‐474 cells (*P* < 0.05 vs. control; Fig. 3A,B), but was not significantly changed in MDA‐MB‐453 cells (Fig. 3A,B), indicating different responses to HP stimulation among cell lines.

**Fig. 2:**
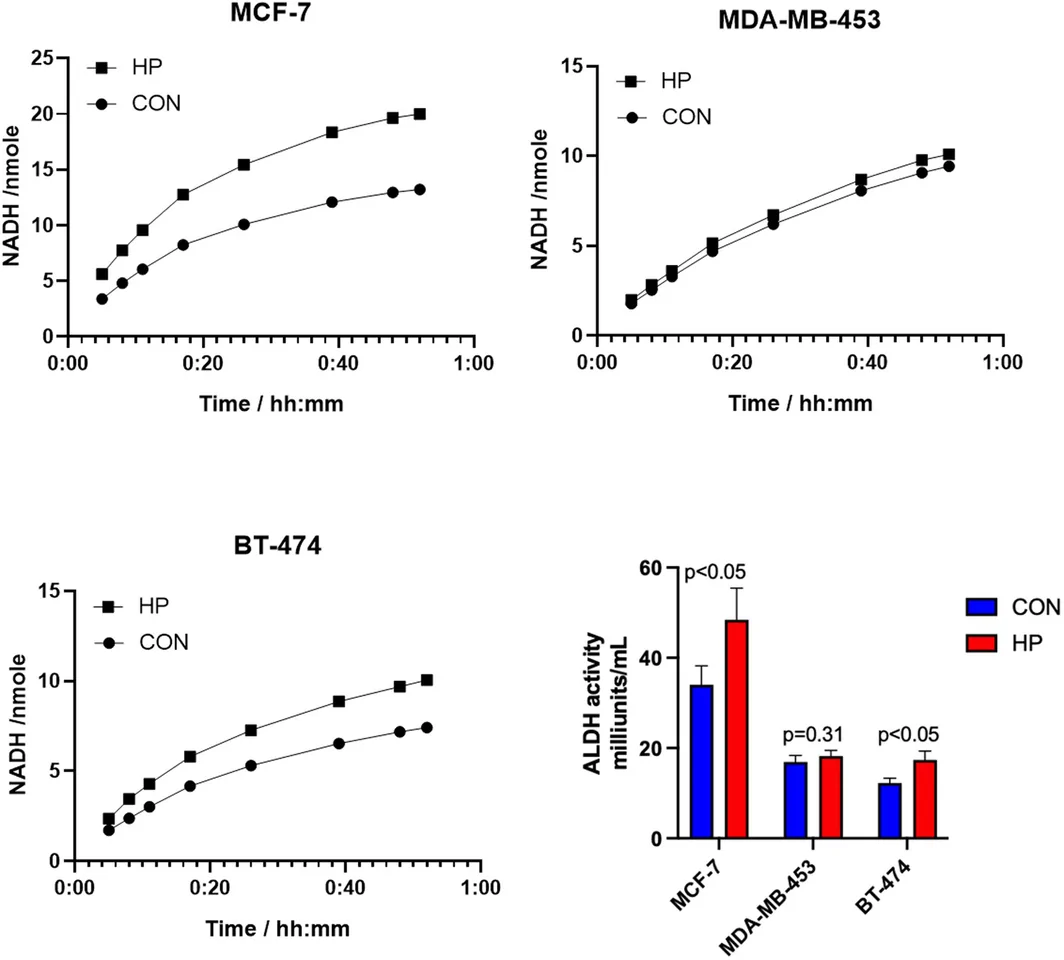
Aldehyde dehydrogenase (ALDH) activity of breast cancer cells following treatment with or without 50 mmHg HP for 48 h. Representative curve of generated NADH amount over time and quantitative data of ALDH activity in MCF‐7, MDA‐MB‐453, BT‐474 cell lines. The data are presented as the mean ± SD from 3 independent experiments. Statistical significance was determined using an unpaired *t*‐test. HP, hydrostatic pressure; CON, control.

**Fig. 3:**
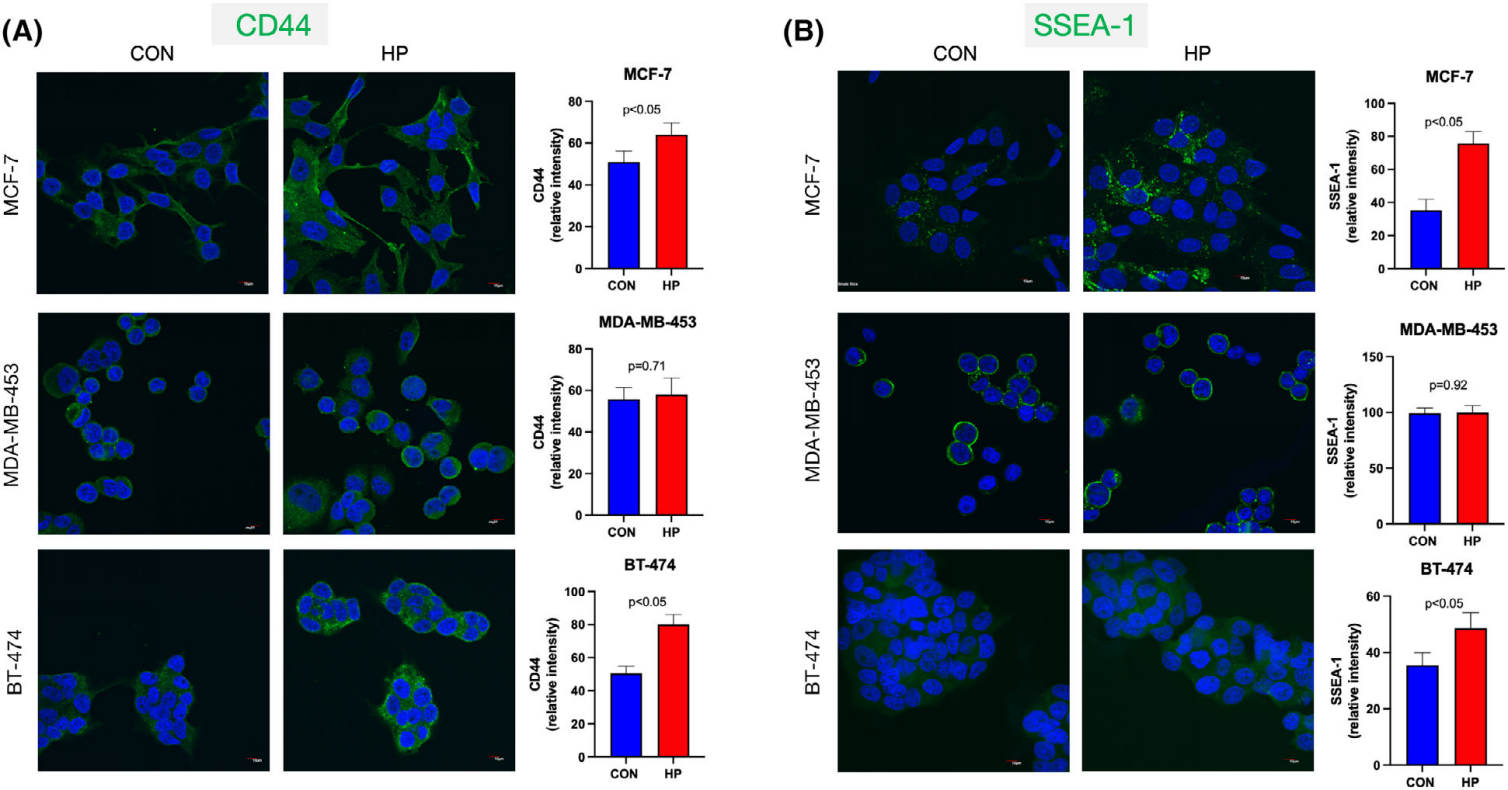
Immunostaining analysis on the expression of stemness markers in breast cancer cells following treatment with or without 50 mmHg HP for 48 h. Representative images (left) and quantitative data (right) on the expression of CD44 (A) and SSEA‐1 (B) in MCF‐7, MDA‐MB‐453, and BT‐474 cell lines. Scale bar, 10 μm. The nuclei were stained with 4, 6‐diamidino‐2‐phenylindole (DAPI). The data are presented as the mean ± SD. Statistical significance was determined using an unpaired *t*‐test. HP, hydrostatic pressure; CON, control.

To understand the molecular mechanism involving the different responses to HP stimulation among three cell lines, we just performed TRITC‐phalloidin staining to detect the actin filaments in cells because mechanical stress is known to induce the formation of actin stress fibers [30]. The MCF‐7 cells and BT‐474 cells formed clearly dense actin fibers (Fig. 4A) and significantly increased fluorescent intensity of staining under 50 mmHg HP stimulation (*P* < 0.05 *vs*. control; Fig. 4B). However, the MDA‐MB‐453 cells showed a round shape and did not induce dense actin fibers (Fig. 4A), and the fluorescent intensity of staining even slightly decreased under 50 mmHg HP stimulation (*P* = 0.27 vs. control; Fig. 4B).

**Fig. 4:**
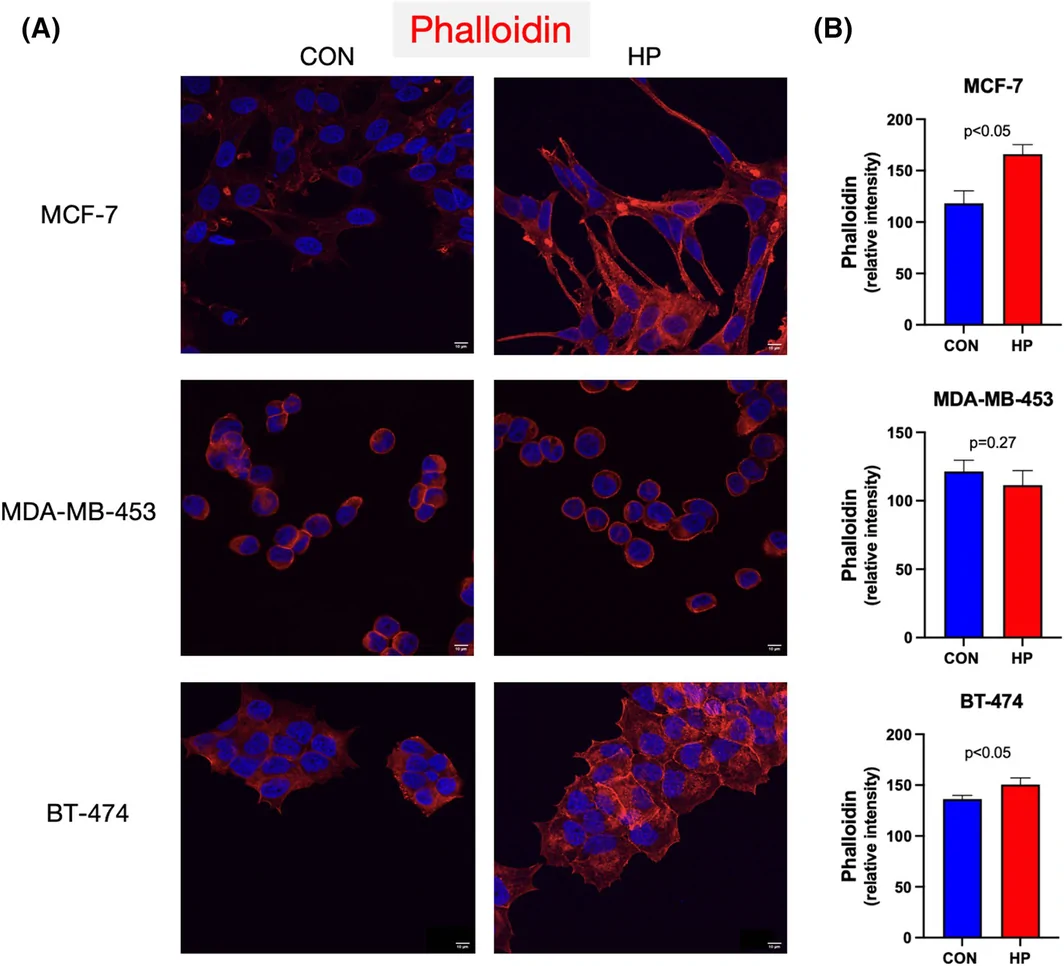
TRITC‐phalloidin staining for Actin filaments of breast cancer cells following treatment with or without 50 mmHg HP for 48 h. Representative images (A) and quantitative data (B) of fluorescence intensity in MCF‐7, MDA‐MB‐453, BT‐474 cell lines. Scale bar, 10 μm. The nuclei were stained with 4, 6‐diamidino‐2‐phenylindole (DAPI). The data are presented as the mean ± SD. Statistical significance was determined using unpaired *t*‐test. HP, hydrostatic pressure; CON, control.

HIF‐1α, a master regulator of the cellular adaptive response to hypoxia, is known to play critical roles in metabolic reprogramming [31] and stemness [4] in cancer cells. Our recent study has found that HP stimulation can enhance the expression of HIF‐1α in mouse Lewis lung cancer cells [28]. Therefore, we measured the expression of HIF‐1α in cells by western blot. We found that 50 mmHg HP stimulation for 48 h significantly enhanced the expression of HIF‐1α in MCF‐7 cells and BT‐474 cells (*P* < 0.05 vs. control; Fig. 5A). However, the expression of HIF‐1α was not detectable in MDA‐MB‐453 cells (Fig. 5A). Therefore, HP stimulation may increase the ALDH activity in breast cancer cells through HIF‐1α signaling.

**Fig. 5:**
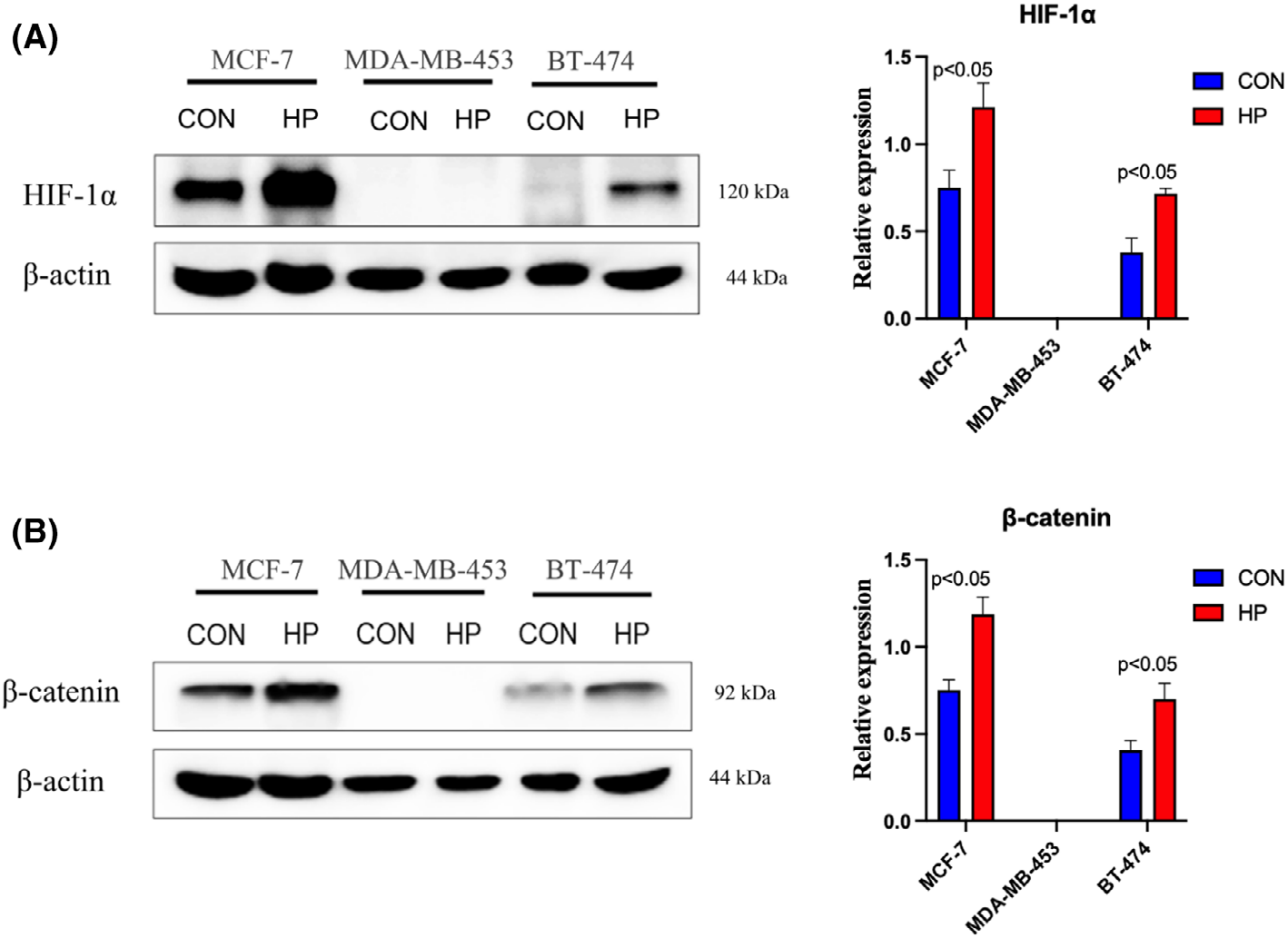
Expression of HIF‐1α and β‐catenin in breast cancer cells. Representative blots and semi‐quantitative analysis on the expression level of HIF‐1α (A) and β‐catenin (B) in cells following treatment with or without 50 mmHg HP for 48 h. The data are presented as the mean ± SD from three independent experiments. Statistical significance was determined using an unpaired *t*‐test. HP, hydrostatic pressure; CON, control.

As an intracellular signal transducer in the Wnt signaling pathway, β‐catenin is reported to be a key regulator and sensor in biomechanical forces [19, 20]. To find the molecular mechanism involving the different responses to HP stimulation among cell lines, we investigated the expression of β‐catenin, a subunit of the cadherin protein complex. As expected, the exposure to 50 mmHg HP for 48 h significantly upregulated β‐catenin in MCF‐7 cells and BT‐474 cells (*P* < 0.05 vs. control; Fig. 5B). Surprisingly, same as HIF‐1α, β‐catenin was also not detectable in MDA‐MB‐453 cells (Fig. 5B). The deficiency of β‐catenin in MDA‐MB‐453 cells has been already reported [32]. Therefore, HP stimulation likely promotes stemness of breast cancer cells via Wnt/β‐catenin and HIF‐1α signaling.

To further confirm/distinguish the role of Wnt/β‐catenin and HIF‐1α signaling in HP‐induced stemness of breast cancer cells, we exposed the cells to 1% O_2_ for 48 h because hypoxia is well known to induce HIF‐1α and stemness. As expected, hypoxia significantly increased the ALDH activity in all three cell lines (*P* < 0.05 vs. control; Fig. 6A), including the β‐catenin deficient MDA‐MB‐453 cell that is unresponsive to HP stimulation. Western blot analysis indicated that hypoxia significantly enhanced HIF‐1α expression in all three cell lines, including the β‐catenin‐deficient MDA‐MB‐453 cell (*P* < 0.05 vs. control; Fig. 6B). Moreover, the expression of β‐catenin in MCF‐7 cells and BT‐474 cells was also significantly upregulated under hypoxia (*P* < 0.05 vs. control; Fig. 6C). These results suggest that Wnt/β‐catenin signaling is necessary for initiating the HP stimulation‐induced stemness of breast cancer cells.

**Fig. 6:**
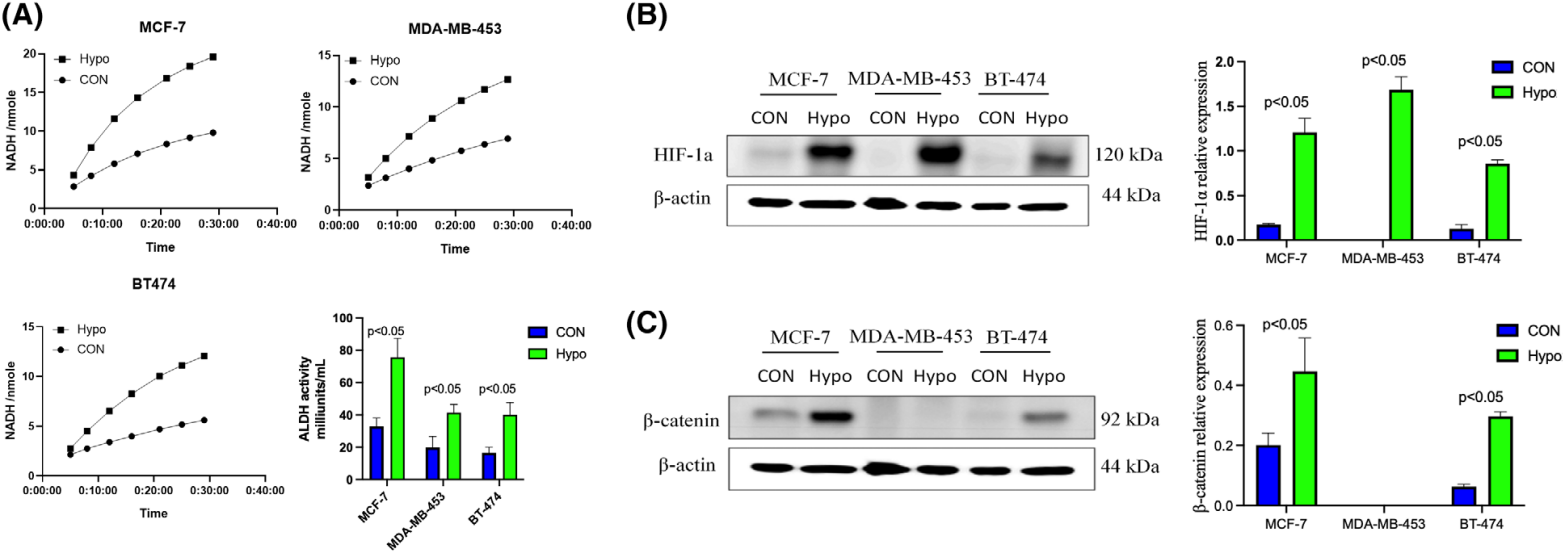
Aldehyde dehydrogenase (ALDH) activity and the expression level of HIF‐1α and β‐catenin in breast cancer cells cultured under 1% O_2_ hypoxia for 48 h. (A) Representative curve of generated NADH amount over time and quantitative data of ALDH activity. Representative blots and semi‐quantitative analysis of the protein expression level of HIF‐1α (B) and β‐catenin (C) in cells. The data are presented as the mean ± SD from three independent experiments. Statistical significance was determined using an unpaired *t*‐test. Hypo, hypoxia; CON, control.

## Discussion

Altered biomechanical forces within the microenvironment of tumor mass have been reported to play critical roles in the progression of malignant tumors [9]. Owing to the hyper‐permeability of immature capillaries, the interstitial fluid HP is commonly increased within malignant tumors [33, 34]. We have recently demonstrated that HP stimulation facilitates cancer cells to acquire premetastatic property [28], but the potential role of elevated HP in promoting stemness has not yet been investigated.

Using three different breast cancer cell lines, we found that *ex vivo* loading of cancer cells to 50 mmHg HP for 48 h significantly increased ALDH activity and enhanced the expression of stemness markers in MCF‐7 cells and BT‐474 cells, suggesting the acquisition of stemness under HP stimulation. However, one cell line (MDA‐MB‐453 cells) did not clearly respond to 50 mmHg HP stimulation at all, including the formation of actin stress fibers and the acquisition of stemness.

Cells sense the surrounding biomechanical forces, such as stiffness and HP by integrins, ion channels and GPCRs [18], and correspondingly change their biological behaviors via mechanotransduction. As one of the major mechanotransduction pathways [19, 20], Wnt/β‐catenin signaling is known to play a critical role in CSC self‐renewal [22], tumor initiation [23], metastasis [35], and chemotherapy resistance [36] in breast cancer. Consistent with the increased ALDH activity and enhanced expression of stemness markers, exposure to 50 mmHg HP clearly upregulated β‐catenin in MCF‐7 cells and BT‐474 cells. However, β‐catenin‐deficient MDA‐MB‐453 cells failed to sensitively respond to 50 mmHg HP stimulation. As one of the major upstream mediators of mechanotransduction pathways, it seems that β‐catenin is critical for initiating the stemness acquisition of cancer cells in response to biomechanical stresses.

Either cyclic or constant HP stimulation has been demonstrated to stabilize HIF‐1α in macrophages and cancer cells [28, 37]. HIF‐1α is well‐known as an important mediator of metabolism reprogramming [38] and stemness formation [31] of cancer cells and is reported to play a pivotal role in promoting specification and maintenance of the CSCs [4, 38]. It has also been reported that HIF‐1α can competitively combine with β‐catenin, forming HIF‐1α/β‐catenin complex and promoting tumor cell proliferation and metastasis [34]. In this study, we found that 50 mmHg HP stimulation increased HIF‐1α expression in MCF‐7 cells and BT‐474 cells, but not in the β‐catenin‐deficient MDA‐MB‐453 cells, suggesting the regulatory role of β‐catenin in HIF‐1α expression induced by mechanical stress. Hypoxia is well known to induce HIF‐1α and stemness. To further distinguish the different roles of HIF‐1α and β‐catenin in stemness induction under HP stimulation, we evaluated the efficiency of stemness induction of three breast cancer cell lines under hypoxia. Correspondence with the upregulated HIF‐1α, hypoxia exposure largely enhanced ALDH activity in all three cell lines, including the β‐catenin‐deficient MDA‐MB‐453 cell. More interestingly, the ALDH activity in the MCF‐7 cells and BT‐474 cells increased to even a much higher level under hypoxia compared with the 50 mmHg HP stimulation, suggesting a higher efficiency of stemness induction under hypoxia. As the increase of ALDH activity is well in parallel with the upregulation of HIF‐1α in all cell lines under either hypoxia or HP stimulation, HIF‐1α may serve as a direct master regulator of stemness induction. However, β‐catenin is necessary for initiating stemness induction under HP stimulation.

According to our experimental data, hypoxia induced stemness (Fig. 6A) with HIF‐1α upregulation (Fig. 6B) in all cell lines, including the β‐catenin deficient MDA‐MB‐453 cells. Hypoxia also upregulated β‐catenin in MCF‐7 and BT‐474 cells (Fig. 6C), suggesting the association between HIF‐1α and β‐catenin. In contrast, HP stimulation upregulated HIF‐1α and induced stemness in MCF‐7 and BT‐474 cells, but not in the β‐catenin deficient MDA‐MB‐453 cells. Differed from hypoxia, a metabolic‐chemical stimulus, primarily stabilizes HIF‐α to promote stemness, it seems that HP activates HIF‐1α via β‐catenin to promote stemness.

The present study has limitations. First, our *ex vivo* experimental approach indicated the HP‐induced stemness features in breast cancer cells; we did not perform *in vivo* experiments for further confirmation. Second, we performed experiments using breast cancer cell lines under monolayer culture conditions rather than using 3D spheroids. Although our experimental data have suggested the potential role of elevated HP in promoting stemness acquisition of cancer cells, it is impossible to completely mimic the *in vivo* intratumoral microenvironment, even when using 3D spheroids for experiments. Third, it can be better to confirm our experimental findings using PDX cells or primary cancer cells from tumor samples of patients. However, cancer cell lines have been commonly used, especially for proof‐of‐concept studies because of their easy accessibility and high stability. Moreover, our data showed the critical role of β‐catenin, probably as the key mechanotransducer in HP‐induced stemness of cancer cells; we did not investigate the downstream mechanotransduction signaling. Otherwise, intervention experiments (β‐catenin loss‐of‐function/rescue and HIF‐1α inhibition) are also required for definitive causal validation of our findings.

In conclusion, our experimental data suggest that mechanical stress, such as the elevation of interstitial fluid HP in the tumor microenvironment may induce HIF‐1α expression via β‐catenin to initiate stemness features of cancer cells (Fig. 7). However, the hypoxic microenvironment in malignant tumors can induce both β‐catenin and HIF‐1α to accelerate the stemness acquisition of cancer cells (Fig. 7). As mechanical stress such as elevated fluid HP in malignant tumors can lead to poor microcirculation to exacerbate blood supply, targeting particular mechanotransduction molecule(s) will be a potential therapeutic approach for CSCs in breast cancers.

**Fig. 7:**
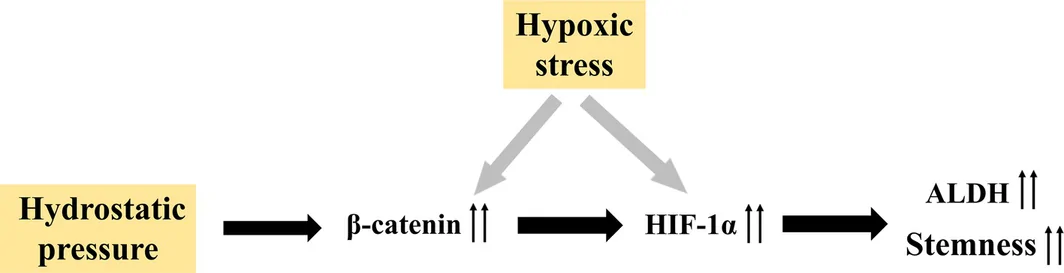
Schematic diagrams of mechanisms on stemness of cancer cells induced by mechanical stress or hypoxic stress.

## Conflict of interest

The authors declare that they have no competing interests.

## Author contributions

DZ, YX, and RD performed the experiments and acquired the data. TSL, DZ, YX, JM, and CY analyzed and interpreted the data. DZ and BJ drafted the manuscript. TSL, JL, and HW critically revised the manuscript for important intellectual content. TSL and DZ confirmed the authenticity of all the raw data. All the authors contributed to the conception and design of the study and read and approved the final version of the manuscript.

## Data Availability

All data supporting the findings of this study are included in this manuscript. The corresponding author, upon request, could share detailed information about this study.

## References

[1] Wu J , Zhu P , Lu T , du Y , Wang Y , He L , Ye B , Liu B , Yang L , Wang J *et al*. (2019) The long non‐coding RNA LncHDAC2 drives the self‐renewal of liver cancer stem cells via activation of hedgehog signaling. J Hepatol 70, 918–929. 10.1016/j.jhep.2018.12.015 30582981

[2] Ramos EK , Hoffmann AD , Gerson SL and Liu H (2017) New opportunities and challenges to defeat cancer stem cells. Trends Cancer 3, 780–796. 10.1016/j.trecan.2017.08.007 PMC5958547 29120754

[3] Ma YS , Yu F , Zhong XM , Lu GX , Cong XL , Xue SB , Xie WT , Hou LK , Pang LJ , Wu W *et al*. (2018) miR‐30 family reduction maintains self‐renewal and promotes tumorigenesis in NSCLC‐initiating cells by targeting oncogene TM4SF1. Mol Ther 26, 2751–2765. 10.1016/j.ymthe.2018.09.006 PMC6277537 30301667

[4] Xiang L and Semenza GL (2019) Hypoxia‐inducible factors promote breast cancer stem cell specification and maintenance in response to hypoxia or cytotoxic chemotherapy. Adv Cancer Res 141, 175–212. 10.1016/bs.acr.2018.11.001 30691683

[5] DeSantis CE , Bray F , Ferlay J , Lortet‐Tieulent J , Anderson BO and Jemal A (2015) International variation in female breast cancer incidence and mortality rates. Cancer Epidemiol Biomarkers Prev 24, 1495–1506. 10.1158/1055-9965.EPI-15-0535 26359465

[6] Clark DW and Palle K (2016) Aldehyde dehydrogenases in cancer stem cells: potential as therapeutic targets. Ann Transl Med 4, 518. 10.21037/atm.2016.11.82 PMC5233526 28149880

[7] Kao YC , Jheng JR , Pan HJ , Liao WY , Lee CH and Kuo PL (2017) Elevated hydrostatic pressure enhances the motility and enlarges the size of the lung cancer cells through aquaporin upregulation mediated by caveolin‐1 and ERK1/2 signaling. Oncogene 36, 863–874. 10.1038/onc.2016.255 27499095

[8] Tse JM , Cheng G , Tyrrell JA , Wilcox‐Adelman SA , Boucher Y , Jain RK and Munn LL (2012) Mechanical compression drives cancer cells toward invasive phenotype. Proc Natl Acad Sci U S A 109, 911–916. 10.1073/pnas.1118910109 PMC3271885 22203958

[9] Bregenzer ME , Horst EN , Mehta P , Novak CM , Repetto T and Mehta G (2019) The role of cancer stem cells and mechanical forces in ovarian cancer metastasis. Cancer 11, 1008. 10.3390/cancers11071008 PMC6679114 31323899

[10] Less JR , Posner MC , Boucher Y , Borochovitz D , Wolmark N and Jain RK (1992) Interstitial hypertension in human breast and colorectal tumors. Cancer Res 52, 6371–6374. 1423283

[11] Nathan SS , DiResta GR , Casas‐Ganem JE , Hoang BH , Sowers R , Yang R , Huvos AG , Gorlick R and Healey JH (2005) Elevated physio‐logic tumor pressure promotes proliferation and chemosensitivity in human osteosarcoma. Clin Cancer Res 11, 2389–2397. 10.1158/1078-0432.CCR-04-2048 15788690

[12] Zanotelli MR and Reinhart‐King CA (2018) Mechanical forces in tumor angiogenesis. Adv Exp Med Biol 1092, 91–112. 10.1007/978-3-319-95294-9_6 PMC6986816 30368750

[13] Gutmann R , Leunig M , Feyh J , Goetz AE , Messmer K , Kastenbauer E and Jain RK (1992) Interstitial hypertension in head and neck tumors in patients: correlation with tumor size. Cancer Res 52, 1993–1995. 1551128

[14] Huang C and Ogawa R (2012) Effect of hydrostatic pressure on bone regeneration using human mesenchymal stem cells. Tissue Eng Part A 18, 2106–2113. 10.1089/ten.TEA.2012.0064 22607391

[15] Luo L , Foster NC , Man KL , Brunet M , Hoey DA , Cox SC , Kimber SJ and el Haj AJ (2022) Hydrostatic pressure promotes chondrogenic differentiation and microvesicle release from human embryonic and bone marrow stem cells. Biotechnol J 17, e2100401. 10.1002/biot.202100401 34921593

[16] Cheng CC , Chung CA , Chang CJ , Cheng YC , Huang CJ , Chien CC and Lin HT (2022) Hydrostatic pressure facilitates calcium deposition and osteogenic gene expression in the osteoblastic differentiation of placenta‐derived multipotent cells. Taiwan J Obstet Gynecol 61, 270–276. 10.1016/j.tjog.2022.02.014 35361387

[17] Li Y , Zhou J , Yang X , Jiang Y and Gui J (2016) Intermittent hydrostatic pressure maintains and enhances the chondrogenic differentiation of cartilage progenitor cells cultivated in alginate beads. Dev Growth Differ 58, 180–193. 10.1111/dgd.12261 26771816

[18] Suresh S (2007) Biomechanics and biophysics of cancer cells. Acta Biomater 3, 413–438. 10.1016/j.actbio.2007.04.002 PMC2917191 17540628

[19] Wang J , Jiang J , Yang X , Zhou G , Wang L and Xiao B (2022) Tethering piezo channels to the actin cytoskeleton for mechanogating via the cadherin‐β‐catenin mechanotransduction complex. Cell Rep 38, 110342. 10.1016/j.celrep.2022.110342 35139384

[20] Zhou T , Gao B , Fan Y , Liu Y , Feng S , Cong Q , Zhang X , Zhou Y , Yadav PS , Lin J *et al*. (2020) Piezo1/2 mediate mechanotransduction essential for bone formation through concerted activation of NFAT‐YAP1‐ß‐catenin. Elife 9, e52779. 10.7554/eLife.52779 PMC7112954 32186512

[21] Astudillo P and Larraín J (2014) (2014) Wnt signaling and cell‐matrix adhesion. Curr Mol Med 14, 209–220. 10.2174/1566524014666140128105352 24467207

[22] Tang T , Guo C , Xia T , Zhang R , Zen K , Pan Y and Jin L (2019) LncCCAT1 promotes breast cancer stem cell function through activating WNT/β‐catenin signaling. Theranostics 9, 7384–7402. 10.7150/thno.37892 PMC6831302 31695775

[23] Lopez AL , Sebbagh M , Bertucci F , Finetti P , Wicinski J , Marchetto S , Castellano R , Josselin E , Charafe‐Jauffret E , Ginestier C *et al*. (2018) The SCRIB paralog LANO/LRRC1 regulates breast cancer stem cell fate through WNT/β‐catenin signaling. Stem Cell Reports 11, 1040–1050. 10.1016/j.stemcr.2018.09.008 PMC6234904 30344009

[24] Brown JM and Wilson WR (2004) Exploiting tumour hypoxia in cancer treatment. Nat Rev Cancer 4, 437–447. 10.1038/nrc1367 15170446

[25] Kheshtchin N , Arab S , Ajami M , Mirzaei R , Ashourpour M , Mousavi N , Khosravianfar N , Jadidi‐Niaragh F , Namdar A , Noorbakhsh F *et al*. (2016) Inhibition of HIF‐1α enhances anti‐tumor effects of dendritic cell‐based vaccination in a mouse model of breast cancer. Cancer Immunol Immunother 65, 1159–1167. 10.1007/s00262-016-1879-5 PMC11029746 27497816

[26] Schito L and Semenza GL (2016) Hypoxia‐inducible factors: master regulators of cancer progression. Trends Cancer 2, 758–770. 10.1016/j.trecan.2016.10.016 28741521

[27] Oskarsson T , Batlle E and Massagué J (2014) Metastatic stem cells: sources, niches, and vital pathways. Cell Stem Cell 14, 306–321. 10.1016/j.stem.2014.02.002 PMC3998185 24607405

[28] Zhai D , Xu Y , Abdelghany L , Zhang X , Liang J , Zhang S , Guo C and Li TS (2021) Hydrostatic pressure stabilizes HIF 1α expression in cancer cells to protect against oxidative damage during metastasis. Oncol Rep 46, 211. 10.3892/or.2021.8162 34368876

[29] Urata Y , Goto S , Luo L , Doi H , Kitajima Y , Masuda S , Ono Y and Li TS (2014) Enhanced Nox1 expression and oxidative stress resistance in c‐kit‐positive hematopoietic stem/progenitor cells. Biochem Biophys Res Commun 454, 376–380. 10.1016/j.bbrc.2014.10.090 25451257

[30] Hoffman LM , Smith MA , Jensen CC , Yoshigi M , Blankman E , Ullman KS and Beckerle MC (2020) Mechanical stress triggers nuclear remodeling and the formation of transmembrane actin nuclear lines with associated nuclear pore complexes. Mol Biol Cell 31, 1774–1787. 10.1091/mbc.E19-01-0027 PMC7521858 31967947

[31] Semenza GL (2009) Regulation of cancer cell metabolism by hypoxia‐inducible factor 1. Semin Cancer Biol 19, 12–16. 10.1016/j.semcancer.2008.11.009 19114105

[32] Kim SY , Dunn IF , Firestein R , Gupta P , Wardwell L , Repich K , Schinzel AC , Wittner B , Silver SJ , Root DE *et al*. (2010) CK1epsilon is required for breast cancers dependent on beta‐catenin activity. PLoS One 5, e8979. 10.1371/journal.pone.0008979 PMC2813871 20126544

[33] Jain RK , Martin JD and Stylianopoulos T (2014) The role of mechanical forces in tumor growth and therapy. Annu Rev Biomed Eng 16, 321–346. 10.1146/annurev-bioeng-071813-105259 PMC4109025 25014786

[34] Senger DR , Galli SJ , Dvorak AM , Perruzzi CA , Harvey VS and Dvorak HF (1983) Tumor cells secrete a vascular permeability factor that promotes accumulation of ascites fluid. Science 219, 983–985. 10.1126/science.6823562 6823562

[35] Lv C , Li F , Li X , Tian Y , Zhang Y , Sheng X , Song Y , Meng Q , Yuan S , Luan L *et al*. (2017) MiR‐31 promotes mammary stem cell expansion and breast tumorigenesis by suppressing Wnt signaling antagonists. Nat Commun 8, 1036. 10.1038/s41467-017-01059-5 PMC5648844 29051494

[36] Sakunrangsit N and Ketchart W (2019) Plumbagin inhibits cancer stem‐like cells, angiogenesis and suppresses cell proliferation and invasion by targeting Wnt/β‐catenin pathway in endocrine resistant breast cancer. Pharmacol Res 150, 104517. 10.1016/j.phrs.2019.104517 31693936

[37] Solis AG , Bielecki P , Steach HR , Sharma L , Harman CCD , Yun S , de Zoete MR , Warnock JN , To SDF , York AG *et al*. (2019) Mechanosensation of cyclical force by PIEZO1 is essential for innate immunity. Nature 573, 69–74. 10.1038/s41586-019-1485-8 PMC6939392 31435009

[38] Kaidi A , Williams AC and Paraskeva C (2007) Interaction between beta‐catenin and HIF‐1 promotes cellular adaptation to hypoxia. Nat Cell Biol 9, 210–217. 10.1038/ncb1534 17220880

